# Prevalence of anaemia among Quranic school (Khalawi) students (Heiran)in Wad El Magboul village, rural Rufaa, Gezira State, Central Sudan: a cross sectional study

**DOI:** 10.11604/pamj.2016.24.244.8355

**Published:** 2016-07-15

**Authors:** Mohammed Saeed Elsamani Eltayeb, Awad Eseed Elsaeed, Ahmed Abdalla Mohamedani, Abbas Abdalrahman Assayed

**Affiliations:** 1 University of Albaha, Saudi Arabia; 2 Faculty of Medicine, University of Gezira, Saudi Arabia

**Keywords:** Anemia among childen, causes, traditional Quranic, schools Khalwa students, iron deficiency anemia

## Abstract

**Introduction:**

This is a cross sectional descriptive community-based study. The aim was to assess the prevalence of anaemia among quranic schoolchildren in khalawi Wad EL Magboul village, rural Rufaa, Gezira State, central Sudan.

**Methods:**

A sample of 180 male participants were included in the study. Informed consent was obtained. Venous blood samples were obtained to measure the hematological parameters and blood films for malaria parasites. Urine and stool analyses were also done. Data were analyzed using SPSS.

**Results:**

The mean age of participants was 12.31 years (SD +/- 2.26). The mean Hb value was 11.75g/dl and it was statistically significant correlation when compared with the mean Hb reference value (13.5g/dl) P value 0,000 (95% CI). Regarding period of stay in the khalwa up to the time of the study, 88 (49.28%) for one year, 54 (30.24%) for 2 years, 22 (12.32%) for 3 years and 16 (8.96%) for more than 3 years. About 77 students (42.78%) were pale on clinical examination. The Mean Cell Hemoglobin (MCH) mean value was 25.58 pg ( 3.55). Many conditions known to be associated with anemia were found; 49 students (27.2%) had a positive blood films for falciparum malaria, 14 students (7.8%) were found to have haematuria and ova of S. haematobium, In169 students (93.4%) stool examination was negative , while 11 students (6.6%) had intestinal worms (Enterobius vermicularis).

**Conclusion:**

Majority of the study participants had iron deficiency anaemia, followed by haemolytic, macrocytic and sickle cell anaemia. This might have negative health and educational implications.

## Introduction

Khalawi (plural of Khalwa) are traditional religious schools, they are very popular in Sudan. The Khalawi (Quran Schools) and Maseeds (Mosques) played and are still playing great roles in designing the consciences of the people of Sudan. They were the available institutions where people learnt teachings of Islam as well as the formal education. Most of them were established by Shiekhs and well-wishers. That distinguish those institutions is that most of them were established in remote and isolated spots of land where no one wanted to live. The idea behind that choice was that those pious men wanted quiet and calm areas so that they could devote most of their time for worshiping and learning. As time passed, those remote and holy places turned into towns and cities [1]. Khalawi funding depends mainly on Islamic endowments. Also they receive donations from philanthropists who believe in the role of being alone in maintaining the book of Allah (Quran) in the hearts of the people of Sudan. There is no fixed period or duration for the study in khalwa (singular), although the period usually ranges between six and eight years, from the time of the enrollment of the child. Children as young as five years may be accepted [2]. The main food for the students or ”Heiran” is a kind of base made from sorghum flour mixed and stirred in a hot water making a hard porridge-like recipe named “Asida”. Then they eat it with a relish or beans [2]. As extra class activities, Heiran are also involved in agricultural activities during the rainy season so that they secure their own food for the rest of the year. Students live in hostels attached to the Maseeds. In the hostels, students are distributed in such a way ensures that each age group live together. Students have to study on everyday basis from dawn until noon when they take a period for rest and eating before resuming their studying again for some hours in the later afternoon hours or early evening. Upon successfully finishing his studies, which is memorization of the holy Quran, the student will be given a certificate. Outstanding and talented students will be given a chance to remain and teach the juniors. Somehow this is like a teaching assistant system. On the other hand there is some sort of corporal punishment for neglectant students [2].

Anaemia is defined as a reduction in the haemoglobin concentration of the blood below normal for age and sex. It is the most common disorder of the blood. However, it can include decreased- oxygen binding ability of each haemoglobin molecule due to deformity or lack in numerical development as in some other types of haemoglobin deficiency. Anaemia is a public health problem that affects populations in both developing and developed countries. Its primary cause is iron deficiency, but a number of other conditions, such as malaria, parasitic infestation, other nutritional deficiencies, and haemoglobinopathies are also responsible, often in combination. It occurs with major consequences for human health as well as social and economic development, and it is an indicator of both poor nutrition and poor health. Measuring Hb is frequently used as a proxy indicator of iron deficiency. The World Health Organization (WHO) has suggested levels of haemoglobin below which anaemia is said to be present. These levels are < 11g/dL (110 g/L) in children aged 1-2 years and < 11.2g/dL (112g/L) in children aged 3-5 years and less than 13.5g/dl in children aged 6-12 years.

Khalawi students are a heterogeneous group with different ethnic and sociocultural backgrounds. This study will open up a venue for other health-related studies to explore and evaluate such learning environment. There are many such khalawi in Sudan which might benefit from this study. The general objective was to determine the prevalence of anaemia among Quranic school students in Wad El Magboul Khalawa in Eastern Gezira state in the period from December 2012 - July 2013. The specific objectives were to: determine the prevalence of anaemia among; identify the common types of anaemia; identify the mean Hb level; identify the possible common cause(s) of anaemia among the study group.

## Methods

**Study design:** A cross-sectional analytical community based study.

**Study area:** The study was carried out in Gezira state, Wad EL Magboul village, Quranic schools (Khalawi). Wad EL Magboul is a small village about 15 km to the south east of Rufaa town. Wad EL Magboul Khalawi are considered as one of the popular Khalawi, not only in Gezira state, but also in Sudan because the students or (Heiran) usually come from different places of Sudan. The Khalawi hostels consist of 15 rooms, each room contains from 5 - 10 students being from the same area or region. From 1925 to 1960 these Khalawi were built in Al dweneeb village where several small Khalawi were branched from there and since 1960 the Khalawi were transferred to Wad EL Magboul in Rufaa rural locality where the current Khalawi are now exist. from different places and of different ages. Two diets meals are served, breakfast and dinner which are made mainly from dura porridge (Assida) with a curry made from lentils or beans.

**Study Population:** Quranic school students (Heiran) studying in Khalawi Wad El Magboul. The total number of students (Heiran) at the time of the study was about 180 students.

**Inclusion criteria:** Being male, any student in Khalawi Wad El Magboul lived at least for one year, age between 8 to 18 years.

**Exclusion criteria:** Students living at home but studying in khalawi Wad El Magboul; Those who are receiving treatment to any type of anaemia or taking hematinics as prophylaxis; Any student more than 18 years or less than 8 years old; Those who lived for less than a year.

**Sampling :** A comprehensive convenient sampling method (total coverage) was adopted. A total of 180 quranic male students (Heiran) were studied.

**Blood samples taking and preparations:** All the 180 students were screened for complete blood count (CBC); peripheral blood smears and reticulocyte count. They also were screened for serum ferritin, BFFM, urine and stool analysis. This analysis was conducted at the medical laboratory, faculty of medicine, university of Gezira, departments of haematology and microbiology. 5 ml of venous blood sample were collected from an antecubital vein by a 5ml syringe from each student and divided in EDTA and plain tubes to perform CBC, peripheral blood smears, reticulocyte count, thick film for malaria and serum ferritin level. The site of collection was cleaned using 70% alcohol and left to dry. An elastic tourniquet was applied to the arm for a period not exceeding one minute to avoid haemoconcentration. 2.5 ml of blood was taken into a container with 0.05ml (K2 EDTA) as an anticoagulant with a concentration of 1.5- 2.2 mg/ml and then the sample gently mixed. 2 ml of blood was delivered in a plain tube, after clot, and then centrifuged; the serum was taken to another plain tube to perform serum ferritin level. Urine and stool samples were also taken in appropriate plastic containers to perform urine and stool analysis. The blood samples were tested within 2 hours of sample collection using an automated blood cell counters (sysmex KN21 analyzer) with a flow cytometry using a laser light to perform full blood count: white blood cell counts (WBCs), red blood cell counts (RBCs), haemoglobin concentration (Hb), hematocrit (Hct), mean corpuscular volume(MCV) , mean corpuscular haemoglobin (MCH) , mean corpuscular haemoglobin concentration (MCHC), and platelet counts (PLTs). It is calibrated by a standardized commercially prepared calibrators. The World Health Organization (WHO) has suggested levels of haemoglobin below which anaemia is said to be present. These levels are < 11g/dL (110 g/L) in children aged 1-2 years and < 11.2g/dL (112g/L) in children aged 3-5 years and less than 13.5g/dl in children aged 6-12 years [3].

**Making a blood film:** Preparation of thin blood films was performed. The frosted glass slides were cleaned free of grease. A drop of blood was placed near frosted end of the slide and a cover glass spreader was applied at an angle of 45 degrees, in front of the drop of blood, and moved making a thin blood film and was then allowed to dry. Then they were labeled with the participant’s number and date of sample collection. The films were then fixed in absolute methanol for 10- 20 minutes. The films were placed horizontally on the staining rack and flooded with Leishman's stain and left for 5 minutes. A double volume of buffer 7.2 was added with gentle blowing over the surface without touching the film surface. The films were left for another 8 minutes and then washed off with buffered 7.2. The back of the slide was cleaned using cotton dipped in alcohol and then left to dry. The same method of preparing the blood film for red cell morphology is used for preparing films for reticulocyte count but the stain used here was methylene blue: 2 or 3 drops of the dye solution were delivered into a 75 x 10 mm plastic tube by means of a plastic Pasteur pipette. 2-4 volumes of the patient’s EDTA-anticoagulated blood were added to the dye solution and mixed. The mixture was kept at 37°C for 15-20 min. The red cells were resuspended by gentle mixing and films were spread on glass slides in the usual way, then they were examined when dried without fixing or counterstaining. An area of film was chosen for the count where the cells are undistorted and where the staining is good. The x100 oil-immersion objective lens was used to count the cells. At least 100 reticulocytes had been counted and at least 10 fields were examined to determine the average number of red cells per field.

**Examination of the blood films:** The identification of the specimen was checked and matched with the corresponding full blood count (FBC) form. The films were examined macroscopically to confirm adequate spreading followed by microscopic examination. A low power field (10 objective) was used to assess the quality of the stain and a (40 objective) to determine the suitable area for blood film examination. The differential white blood counts were performed manually using the oil emersion lens. At least one 100 cells were examined. The manual differential white blood cells were compared with the automated differential white blood cells. The morphology of the red cells regarding the staining character, shape, size of the cells and the presence of nucleated red blood cells was recorded. The platelets were examined and estimation of their number, size, morphology and presence of aggregates were commented on.

**Determination of serum ferritin:** Serum ferritin was determined manually using colorimeter by adding 2 volumes (2 ml) of reagent A to 1 volume (1ml) of reagent B (working reagent). Then the working reagent and the instrument were brought to 37°C. Zeroing of the instrument by distilled water was done. Then 1ml from the working reagent to 30µl of the sample was pipetted into a cuvette which was mixed and inserted into the instrument and the stopwatch was started. Then the absorbance at 540 nm after 10 seconds (A_1_) and after 5 minutes (A_2_) was recorded.

**Thick films for malaria:** Thick blood films for malaria were also made and stained with the standard Giemsa, and then microscopically examined using oil immersion.

**Urine analysis:** Explanation to the patient for the need to collect the urine was done. The containers were labeled with the date, the name and number of the patient, and the time of collection. As soon as possible, the specimen was delivered. Urine for sugar and acetone was examined by the dipstick method, then about 5 ml of well mixed urine aseptically was transferred to a labeled conical tube and was centrifuged at 500-1000 g for 5 minutes. The supernatant fluid (by completely inverting the tube) was poured into a second container not the original one. Then, a wet preparation from the remaining sediment was done and microscopically was examined using the x10 and x 40 objective.

**Stool analysis:** Stool analysis was done and the appearance of the specimen has been described (colour of the specimen, whether it is formed, semiformed, unformed or fluid, the presence of blood, mucus or pus, the presence of worms, e.g.(Enterobius vermicularis, Ascaris lumbricoides, or tapeworm segment, e.g. Taenia species). Then a drop of fresh physiological saline on one end of a slide was placed, and a small amount of fresh specimen was mixed with the drop of saline. Then the preparation was covered with a cover glass and microscopically was examined using the x10 and x 40 objectives with the condenser iris closed sufliciently to give good contrast. The specimen was examined for motile E. histolytica trophozoites containing red cells, motile G. lamblia trophozoites, motile Strongyloides larvae, and the eggs and cysts of parasitic pathogens.

**Statistical analysis:** The results were analyzed using statistical software package of social sciences (SPSS) version 16 and descriptive data were expressed as means and percentages with **P** value 0,05. 95% confidence interval of the difference **Chi** square test = 1.57.

**Ethical clearance:** Ethical approval was obtained from the University of Gezira ethical committee and permissions from the medical laboratory and khalawi Wad EL Magboul authority. Target population was informed about the study objectives and was consented prior filling questionnaires. Confidentiality and privacy of target population was guaranteed.

## Results

### The participants’ characteristics

#### Age distribution

A total of 180 subjects, aged between 8-18 years with a mean age of 12.31 years (SD +/- 2.26) were enrolled in the study.

#### Tribal distribution

**Figure 1:**
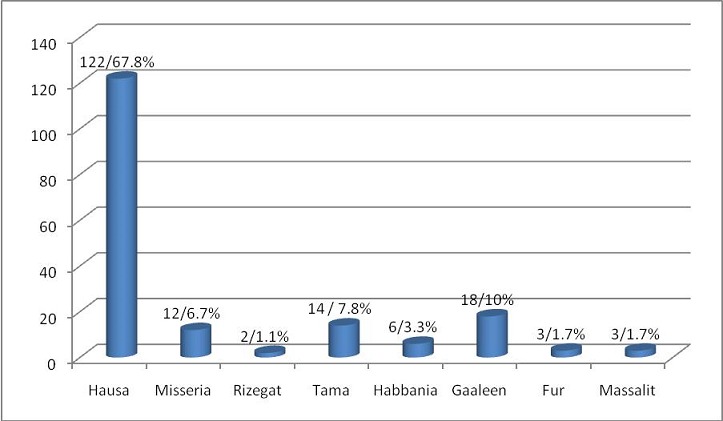
Tribal distribution (n= 180)

The study group subjects were mainly from Hausa (67.8%), next to that was Gaaleen (10%), Tama (7.8%), Misseria (6.7%), Habbania (3.3%), Fur (1.7%), Massalit (1.7%) and Rizegat (1.1%) (Figure 1).

### Other participants’ characteristics

All the students were from the rural areas. Of them 141 students (84.6%) were found to be from poor families, while 39 students (15.4%) belonged to families of moderate socioeconomic status. About 136 students (75.56%) were coming from families consisting of 5- 10 members, while 36 students (20%) came from families consisting of more than 10 members and only 8 students (4.44%) of families consisting of less than 5 members. The type of diet for all the students didn’t contain meat or vegetables, because they eat the same type of food every day (twice a day) which contains mainly dura and beans. The monthly pocket money for 150 students (83.34%) was found to be irregular, while only 30 students (16.66%) had their adequate money. Regarding period of stay in the khalwa up to the time of the study, 88 (49.28%) for one year, 54 (30.24%) for 2 years, 22 (12.32%) for 3 years and 16 (8.96%) for more than 3 years. During this period, the frequency of home visits was variable. The home visit for 161 students (89.16%) was once a year, and they stay there for an average of one month. While the home visit for 15 students (8.4%) was 3 monthly during which they stayed for 1- 2 weeks. Only 4 students (2.24%) had monthly visits to their home and most of them stay for about one week. The knowledge about anaemia among the students was varying. Some students 23/180 (12.88%) had some knowledge about anaemia as they are having anaemia before. About 49students (27.44%) know about but didn’t have anaemia before, while 108 students (60.48%) had no knowledge about anaemia at all. The majority of the students 177/180 (98.2%) had no history of chronic disease, while 3 students (1.8%) were found to have a positive history of recurrent mild epistaxis. The majority of the students, (161, 89. 44%) were not admitted to hospital at all, while 19 students (10.56%) were hospitalized with different diseases: malaria, 10 students (5.6%), 2 students (1.11%) had a history of hospitalization due to acute appendicitis and 3 students (1.67%) due to gastroenteritis, while 4 students (2.22%) due to Schistosomiasis.

### Clinical examination

Clinical examinations revealed pallor in 77 students (42.78%). None of the students had organomegaly or jaundice. Also there were no hair, eye, mouth or nail changes detected among the participants.

### Investigations

Blood films for malaria showed that 49 students (27.2%) had a positive blood films for falciparum malaria. Urine analysis revealed that 164 students (91.1%) had clear urine, 14 students (7.8%) were found to have haematuria and ova of S.haematobium, while 2 students (1.1%) were found to have pyuria. In169 students (93.4%) stool examination was negative, while 11 students (6.6%) had intestinal worms (Enterobius vermicularis) Table 1.

**Table 1:** BFFM, Urine & Stool analysisfor study group subjects (n = 180)

Type of investigation	Frequency	%
BFFM (+)ve	49	27.2
BFFM (-) ve	131	78.8
Urine analysis clear	164	91.1
Haematuria &ova S.haematobium	14	7.8
Pyuria	2	1.1
Stool analysis clear	169	93.4
Worms	11	6.6

### The White cell count and morphology

The mean white blood cell counts value was found to be 5.19 x 10 ^3^ /µl +/- 1.52 standard deviation, with a minimum value of 1.60 x 10^3^/µl and maximum value 14.30 x 10^3^/µl. 93.9% (169 cases) had normal WBCs between 3-9x10^3^ Table 2. Regarding WBCs morphology 152 participants (84.12%) had normal morphology, 14 cases (7.84%) had eosinophilia, 5 cases (2.8%) had hypersegmented neutrophils.8 cases (5.04%) had reactive and atypical lymphocytes, and one case (0.56%) showed few lymphoblasts, but no evidence of marrow failure or organomegaly. A later CBC was done and no blasts were detected so it could be atypical lymphocytes due to viral infection. No myeloblast, promyelocytes, myelocytes, metamyelocytes were detected in the blood films Table 3.

**Table 2:** Descriptive Statistics of CBC Parameters compared with the normal reference values

Parameters	Mean	Std. Deviation	Minimum	Maximum	Normal reference values
WBC counts	5.1918	1.52508	1.60	14.30	4.4 - 10.7 x 10^3^ μ/l
RBC counts	4.5441	.56028	3.10	6.45	4.5 - 5.5 x 10^6^ μ/l
Haemoglobin	11.7572	1.87932	6.00	15.40	11.5 - 15.5 g/dl
PCV	36.5967	3.51979	24.90	45.40	35 - 45 %
M C V	77.8356	8.11169	58.60	101.00	77 - 93 fl
MCH	25.5856	3.55822	17.70	34.10	26 - 34 pg
MCHC	32.9617	2.22566	22.10	36.30	31 - 37 g/dl
Platelets	317.2167	120.60	25.00	723.00	150 - 450 x 10^3^ μ/l
Reticulocytes	1.5544	1.32679	.20	7.00	0.5 - 2.5 %

**Table 3:** White blood cell morphology in thestudy group subjects (n = 180)

White blood cell morphology	Frequency	%
Normal morphology	152	84.12
Eosinophilia	14	7.84
Reactive and a typical lymphocytes	9	5.04
Hypersegmented neutrophils	5	2.8
Total	180	100.0

### Red blood cell count & morphology

The mean RBCs value was found to be 4.54 x 10^6^ /µl +/- 0.560 standard deviation, with a minimum value of 3.10 x 10 ^6^ /µl and maximum value of 6.45 x 10^6^µ/l Table 4.

**Table 4:** RBCs morphology in study group subjects (n = 180)

RBCs morphology	Frequency	%
Anisocytosis, microcytic hypochromic	103	57.68
Normochromic normocytic	49	27.4
Dimorphic blood films	19	10.6
Polychromatic cells	16	8.6
Nucleated red blood cells	14	7.84
Rouleaux formation	9	5.04
Target cells	9	5.04
Macrocytes	7	3.9
Sickle cells	5	2.8

### RBCs morphology

Normochromic normocytic red cells were found in 49 cases (27.4%), dimorphic blood films in19 cases (10.6%), anisocytosis, with predominant microcytic hypochromic cells in 103 cases (57.68%), macrocytes in 7 cases (3.9%), sickle cells in 5 cases (2.8%), target cells in 9 cases (5.04%), polychromatic cells in16 cases (8.96%) nucleated red blood cells in 14 cases (7.84). Marked rouleaux formation was detected in 9 cases (5.04). No autoagglutination was detected in the smears Table 4.

### Haemoglobin level

The mean Hb value was found to be 11.75 g/dl (SD 1.87), with a minimum value of 7.50 g/dl and maximum value of 15.40 g/dl Table 5.

**Table 5:** Haemoglobin level in study group subjects (n = 180)

Haemoglobin values	Frequency	%
< 9 g/dl Severe anaemia	21	11.7
9-13.5 g/dl Mild anaemia	132	73.3
More than 13.5 g/dl	27	15.0
Total	180	100.0

### Packed cell volume (PCV)

The mean PCV value was found to be 36.59% +/-3.51% standard deviation with a minimum value of 24.90% and maximum value of 45.40% Table 2.

### Mean cell volume (MCV)

**Figure 2:**
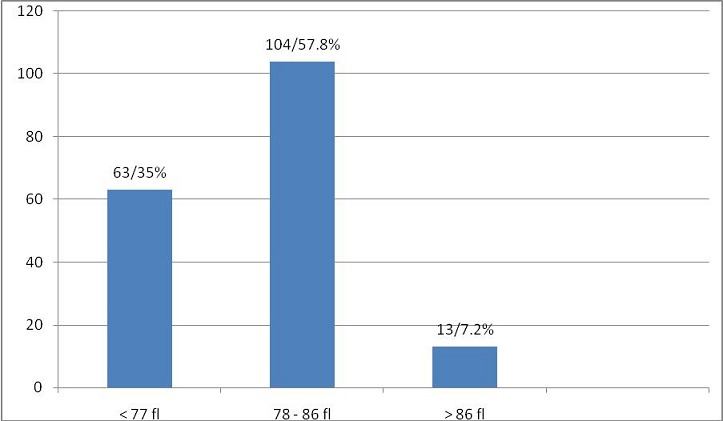
Mean cell volume (n = 180)

The mean MCV value was found to be 77.83 fl +/-8.11standard deviation with a minimum value of 58.60 fl and maximum value of101.00 fl. 104 cases (58.24%) had MCV in the range of 78-86 fl (Figure 2).

### Mean cell haemoglobin (MCH)

**Figure 3:**
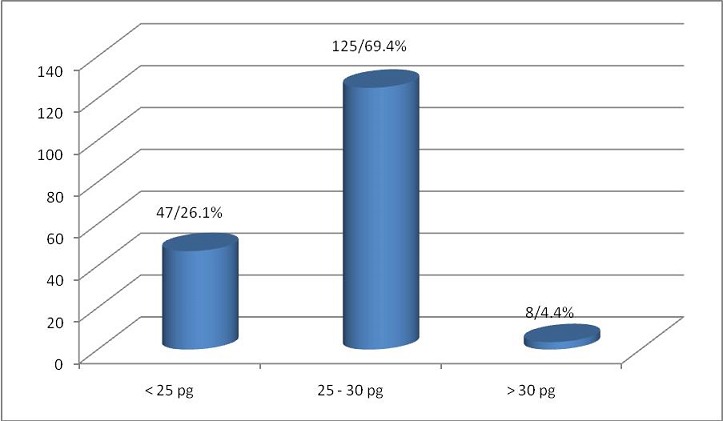
Mean cell haemoglobin (n = 180)

The MCH mean value was found to be 25.58 pg +/-3.55 standard deviation with a minimum value of 17.70 pg and maximum value of 34.10pg. 125 cases (69.4%) had MCV in the range of 25- 30 pg (Table 2) (Figure 3).

### Mean cell haemoglobin concentration (MCHC)

The MCHC mean value was found to be 32.96 g/dl +/- 2.22 standard deviation with a minimum value of 22.10 g/dl and maximum value of 36.30 g/dl Table 2.

### Platelets

The platelets mean value was found to be 317 x 10^3^ /µl +/- 120.60 standard deviation with a minimum value of 25.00 x 10^3^ /µl and maximum value of 723.00 x 10^3^ /µl (Table 2). Most of the students had normal platelet counts, but 5 cases (2.8%), had thrombocytopenia. 25 cases (13.9%) had platelets more than 450 x 10^3^ /µl. Aggregates were found in 52 cases (29.5%). Giant forms were found in 13 cases (7.2%).

### Reticulocyte count

The reticulocyte count mean value was found to be 1.55% +/- 1.32% standard deviation with a minimum value of 0.20% and maximum value of 7%, (Table 2). 38 cases (21.1%) had high reticulocyte counts (due to malaria, schistosomiasis and few other types of haemolytic anaemias (Table 6).

**Table 6:** The reticulocyte count in study group subjects (n = 180)

Reticulocyte counts	Frequency	%
less than 0.5%	72	40.0
0.5 - 2.5%	70	38.9
More than 2.5%	38	21.1
Total	180	100.0

### The Serum ferritin

**Figure 4:**
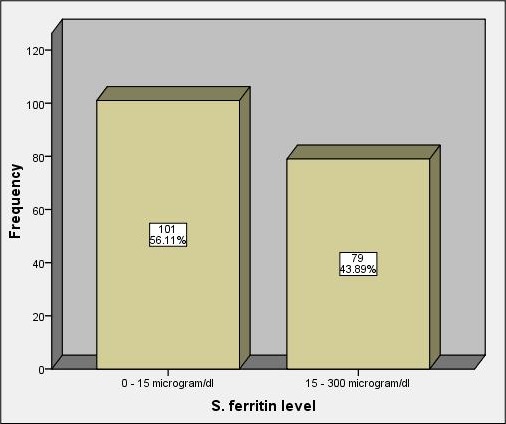
Serum ferritin values (n = 180)

**Figure 5:**
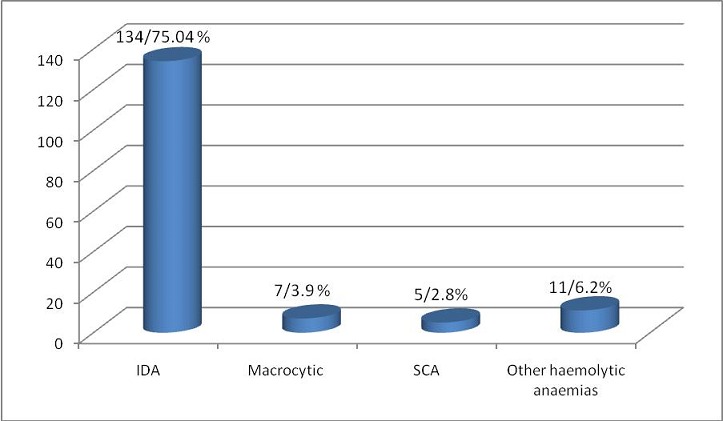
Types of anaemia found in 153 students out of 180 study group subjects

The Serum ferritin mean value was found to be 50.31 µg/l +/-49.58 standard deviation, with a minimum value 0.00 µg/l and maximum value 300.00 µg/l. 101 cases (56.11%) had low ferritin, while none had ferritin >300 µg/l, (Figure 4). According to CBC findings combined with peripheral blood morphology and reticulocyte count, causes and types of anaemia were thought to be iron deficiency anaemia (IDA) in 134 cases (75.04%), macrocytic in 7 cases (3.9%), sickle cell anaemia in 5 cases (2.8%) and other types of haemolytic anaemias in 11 cases (6.2%) (Figure 5). The criteria used to diagnose IDA were low RBCs indices, microcytic hypochromic picture, low reticulocyte counts and low serum ferritin. All cases diagnosed as SCA were from Hausa (3 cases) and Misseria (2 cases) tribes. The presence of polychromasia, NRBCs and high reticulocyte counts without obvious sickle cells made the diagnosis of other haemolytic anaemias more likely, but no further investigations were done. The relationship between mean Hb levels and different types of anaemia found, malaria and S.haematobium infection shown in (Table 7).

**Table 7:** Relationship between mean Hb levels and different types of anaemia found, malaria and S. haematobium infection

Type of anaemia or parasitic infection	No.	Mean Hb level g/dl
Iron deficiency anaemia	134	11.66
Macrocytic anaemia	7	12.45
Sickle cell anaemia	5	12.00
Other haemolytic anaemias	11	12.70
Malaria	49	11.98
S.haematobium infection	14	12.27

## Discussion

Anaemia is a common health problem that affects populations in both developing and developed countries. Its primary cause is iron deficiency, but a number of other conditions, such as malaria, parasitic infection, other nutritional deficiencies, and haemoglobinopathies are also responsible, often in combination. The WHO Global Database on Anaemia can be used to describe the nutritional status of populations and to identify the needs for interventions to prevent and control anaemia [4]. This is probably the first study in this area. All the study group subjects came from rural areas, the majority of them belonged to certain tribes (Misseria, Hausa) who are also known to have congenital anaemias (sickle cell anaemia), and all of them eat the same type of poor diet which consists of dura and beans, so it showed that anaemia should be considered as a major health problem in quranic school (khalawi) children. In the present study, the prevalence of anaemia in Khalawi Wad El Magboul was 88.33%, and the prevalence was as high as 47.11% in children aged 11-14years , 38.15% in children aged between 7-10 years and 3.07% in children aged between 15-18 years. This result is in agreement with one study carried out in Nepal about the prevalence of anaemia amongst adolescents. It was a cross sectional community based study carried out in Morang district to determine the prevalence and distribution of anaemia in terms of age, sex and locations (urban and rural) among adolescent population. Sahli method was used to determine the haemoglobin level. Three hundred and eight adolescents (127 urban, 181 rural in terms of location and 151 male, 157 female in terms of sex) participated in the study. The study found that the overall prevalence of iron deficiency anemia among adolescent population was 65.6% with the distribution of rural 62.4%, urban 70.0%, male 52.3% and female 78.3% [5]. The present study is not in agreement with a study done in Urban Delhi in four primary schools, in which the prevalence of anaemia was 41.8%. This disagreement might be due to differences between the two study areas and the difference socioeconomic status of the two study populations. In Sudan, another parallel study was done about prevalence of anaemia among school children in eastern Sudan, done by Shams E. Musa et.al who found that the prevalence of anaemia in a total of 401 children selected randomly from four randomly selected primary schools in Kassala was 93%. The prevalence of anaemia was estimated, clinically and by measuring haemoglobin concentration [3]. The results showed that clinical examination revealed anaemia in 373 of the students and haemoglobin estimations proved anaemia in 93% of the students enrolled in the study (Hb. less than 13.5 g/dl). Iron deficiency anaemia was found to be the commonest in the present study and this agreed with another study conducted in departments of paediatrics, pathology and pharmacology, University College of Medical Sciences and Guru Teg Bahadur Hospital, Delhi, India, about the Prevalence and etiology of nutritional anaemia among schoolchildren of urban slums [6].

There was significant relation with the mean Hb value of the screened subjects which was found to be 11.75 g/dl (Std. 1.87, minimum value 7.5 g/dl, maximum 15.40g/dl) when compared with the mean Hb reference value (13.5 g/dl) **(P value 0,000. 95% confidence interval of the difference)**. Also there was significant correlation between the low serum ferritin when cross tabulated with the type of diet, **(Chi square test = 1.57, P value = 0.005)**. Seven cases (3.9%) presented with white blood count less than 3 x 10^3^µ/L (leucopenia with neutropenia, associated with reactive lymphocytes) most probably due to chronic or viral infection. White blood count more than 9 x 10^3^µ/L was observed in 4 children (2.2%) dominated by neutrophils which was suggestive of pyogenic infection. Hypersegmented neutrophils were observed in 5 cases (2.8%), suggestive of mixed deficiency. The eosinophils percentage of more than 6% was observed in 14 subjects (7.84%), this eosinophilia probably reflects parasitism (e.g. Schistosomiasis). 5 children (2.8%) presented with platelets counts less than 150 x10^3^µ/l (thrombocytopenia), may be due to asymptomatic malaria parasitisaemia. Aggregates were found in 52 cases (29.5%) and part of this may be due to the presence of inappropriate clots. Thrombocytosis with platelet counts more than 450 occurred in 25 subjects (13.9%) accompanied by low MCV and low MCH (suggestive of iron deficiency) which is one of the causes of thrombocytosis. Very commonly, the platelet count is slightly above the high limits of normal in iron deficiency anemia. This effect was classically postulated to be due to high erythropoietin levels in the body as a result of anemia, cross-reacting to activate thrombopoietin receptors in the precursor cells that make platelets. Such slightly increased platelet counts present no danger, but remain valuable as an indicator of iron deficiency. The likely causes of anaemia in the study may include: nutritional deficiency-major cause; Parasitic infestation (malaria, bilharzia); Rarely hereditary causes.

## Conclusion

The higher prevalence of iron deficiency anaemia, as noticed in the present study, may be attributed to inadequate food intake, poor stores and other nutritional deficiencies among these children. Childhood anaemia continues to be a significant public health problem in school children and iron deficiency either alone or in combination is the commonest nutritional cause of anaemia. Pure macrocytic anaemia in quranic schoolchildren aged 8-11years due to vitamin B12 or folate deficiency is not detected, but there is a possibility of mixed deficiency in this age.

### What is known about this topic

- Students of primary education in the developing countries are prone to anemia.
- The most common type of anemia among primary schools is the “iron deficiency anemia”.

### What this study adds

- This study added that students of informal education “e.g. traditional Quraanic schools” are subject to develop anemia;
- Also there were many associated contributing factors to the development of anemia.
